# Effect of Ambient Bright Light on Behavioral and Psychological Symptoms in People With Dementia: A Systematic Review

**DOI:** 10.1093/geroni/igac018

**Published:** 2022-03-24

**Authors:** Ying-Ling Jao, Julian Wang, Yo-Jen Liao, Jyotsana Parajuli, Diane Berish, Marie Boltz, Kimberly Van Haitsma, Nan Wang, Lauren McNally, Margaret Calkins

**Affiliations:** Ross and Carol Nese College of Nursing, Pennsylvania State University, University Park, Pennsylvania, USA; Department of Architectural Engineering, Pennsylvania State University, University Park, Pennsylvania, USA; Materials Research Institute, Pennsylvania State University, University Park, Pennsylvania, USA; Ross and Carol Nese College of Nursing, Pennsylvania State University, University Park, Pennsylvania, USA; School of Nursing, University of North Carolina at Charlotte, Charlotte, North Carolina, USA; Ross and Carol Nese College of Nursing, Pennsylvania State University, University Park, Pennsylvania, USA; Ross and Carol Nese College of Nursing, Pennsylvania State University, University Park, Pennsylvania, USA; Ross and Carol Nese College of Nursing, Pennsylvania State University, University Park, Pennsylvania, USA; Department of Architectural Engineering, Pennsylvania State University, University Park, Pennsylvania, USA; Ross and Carol Nese College of Nursing, Pennsylvania State University, University Park, Pennsylvania, USA; IDEAS Institute, Cleveland Heights, Ohio, USA

**Keywords:** Alzheimer’s disease, Ambient light, Behavior, BPSD, Dementia

## Abstract

**Background and Objectives:**

Behavioral and psychological symptoms of dementia (BPSD) commonly occur in persons living with dementia. Bright light (BL) interventions have shown some positive impact on BPSD. Ambient lighting is a more efficient approach to delivering BL with better compliance and less staff workload than individual-based lighting interventions. Yet, its effect has not been systematically reviewed. This review synthesized research evidence on the effect of ambient BL on BPSD.

**Research Design and Methods:**

This review searched literature from PubMed (Medline), CINAHL, Scopus, Web of Science, and Cochrane in February 2021. Original research testing the effect of ambient BL on BPSD in persons with dementia was included. Two reviewers independently screened, extracted data, and assessed the quality of each article.

**Results:**

Nine studies were reviewed with 1 randomized controlled trial and 8 quasi-experimental studies. The sample size ranged from 14 to 89 participants across care settings. While not all studies showed positive results, evidence from multiple studies revealed the positive effect of ambient BL on depressive symptoms and agitation in persons with dementia. The ambient BL that showed a positive effect targeted at approximately 350–750 lux, 4,500–9,325 K, and/or circadian stimulus = 0.375–0.4 for 10–12 hr a day for 4 weeks or longer. Evidence on other BPSD was mixed or too limited to draw conclusions.

**Discussion and Implications:**

A preponderance of evidence suggests that, when properly designed and implemented, ambient BL shows promise in reducing depressive symptoms and agitation. Future research, using more rigorous designs, is needed to further test the effect of ambient BL on BPSD with attention to lighting parameters, measurement approaches, and intervention fidelity.

**Translational Significance:** Bright light (BL) interventions have shown some positive impact on behavioral and psychological symptoms of dementia (BPSD). Ambient BL is an effective approach to deliver BL, but its evidence has not been reviewed. This review synthesized evidence on the effects of ambient BL on BPSD. The results revealed that BL interventions targeted at 350–750 lux, 4,500–9,235 K, and/or circadian stimulus = 0.375–0.4 for 10–12 hr a day 4 weeks or longer have positive effects on depressive symptoms and agitation. Ambient BL can be used to improve dementia care and reduce depressive symptoms and agitation in persons with dementia.

## Introduction

### Significance of Behavioral and Psychological Symptoms of Dementia

Up to 97% of persons living with dementia experience behavioral and psychological symptoms of dementia (BPSD; Gruber-Baldini et al., 2004; Ringman & Schneider, 2019). Common BPSD includes agitation, aggression, wandering, apathy, depressive symptoms, and anxiety. BPSD results in significant negative consequences, including functional decline, poor quality of life, and caregiver burden (Gruber-Baldini et al., 2004). Nonpharmacological interventions are recommended as the first-line treatment, but most are labor-intensive and show mixed effects on BPSD (Gruber-Baldini et al., 2004; Mitolo et al., 2018). Therefore, identifying effective and feasible nonpharmacological interventions to reduce BPSD is imperative (Barrick et al., 2010; Ringman & Schneider, 2019). One such promising intervention is changes in lighting conditions in the living environment.

### Impact of Bright Light for Persons with Dementia

Over the past two decades, research has tested the impact of bright light (BL) on BPSD (Missotten et al., 2019). Evidence suggests that poor sleep, circadian disruption, short daylight exposures, and BPSD are associated with interior daylight conditions, with the most robust evidence concerning depressive symptoms and agitation (Figueiro et al., 2014; Goudriaan et al., 2021; Grace, 2002). Persons living with dementia have degenerative changes in the suprachiasmatic nuclei (SCN) of the hypothalamus, which is responsible for generating a circadian rhythm (Figueiro, 2017). This degeneration can deteriorate biological rhythm and contribute to agitation (Figueiro, 2017; Figueiro et al., 2014). Light is the strongest external stimuli regulating the circadian rhythm; yet persons living with dementia, especially those residing in shared residential settings, are not exposed to sufficient sunlight to maintain a stable rhythm (Kim et al., 2003; Konis et al., 2018). Based on this biological basis, BL interventions work to mimic exposure to natural light and regulate SCN, maintain a stable circadian rhythm, and reduce BPSD (Grace, 2002; Van der Ploeg & O’Connor, 2014). Moreover, lighting interventions are noninvasive and have minimal adverse effects (Figueiro, 2017), making them ideal nonpharmacological interventions for persons living with dementia.

### Ambient BL

Despite the positive impact of BL on persons with dementia, such interventions have not been widely implemented in “real world” care settings (Hanford & Figueiro, 2013). Traditional delivery methods use lightboxes that require persons with dementia to sit and keep their eyes oriented toward the BL for 1–2 hr (Kim et al., 2003; Mitolo et al., 2018). However, this approach can encounter compliance issues and add considerable workload to busy staff in shared residential settings. Thus, a different approach to delivering BL is needed. Interest has arisen in providing BL via ambient light (general room illumination) as a part of the built environment (Mitolo et al., 2018). Meanwhile, a growing number of new tunable LED luminaires, using embedded sensors, address implementation barriers and provide new opportunities for auto-controlling indoor electrical and natural light conditions with designed intensity, distribution, and spectra (Van der Ploeg, & O’Connor, 2014). Several studies have evaluated the effect of ambient BL in persons with dementia (Mitolo et al., 2018). Yet, the evidence is inconsistent and the gold standard to design and measure ambient BL has not been established.

### Gaps in Research Evidence of Ambient BL

Grounded in the theoretical basis of the impact of light on circadian rhythm and aging vision, high-intensity lighting and circadian stimulation during the day and low stimulation with less short-wavelength content at night are recommended for persons with dementia (Barrick et al., 2010; Hanford & Figueiro, 2013). This maintains a dark–bright cycle to regulate rest/activity rhythm, reduces sleep disturbance, and consequently reduces BPSD (Figueiro, 2017; Hanford & Figueiro, 2013; Konis et al., 2018). Despite the consensus on the principle of general lighting design, the BL dosages and measures vary widely in current BL intervention research.

A few systematic reviews have been published to report the impact of BL on persons with dementia (Goudriaan et al., 2021; Kim et al., 2003; Mitolo et al., 2018). However, most reviews combined evidence of all kinds of BL with light delivery methods varying from portable desktop-type lightboxes to room-based ambient lighting (Mitolo et al., 2018). In addition, even though the design parameters of existing BL varied widely across studies, they were not explicitly compared in current literature. Thus, establishing precise measures and effective lighting schemes are needed to move forward with BL interventions. To address these gaps, this systematic review aimed to focus on the effect of ambient BL on BPSD and provided an in-depth review of the intervention characteristics (e.g., color, intensity, the timing of the day, light measures, and duration and frequency). This evidence is beneficial to guide clinical practice and future research in designing effective ambient light to reduce BPSD in persons with dementia.

## Method

### Search Strategy and Article Selection

A comprehensive literature search was conducted using PubMed (Medline), CINAHL, Scopus, Web of Science, and Cochrane databases in February 2021. The search was only limited to articles published in English. The literature search was guided by the Preferred Reporting Items for Systematic Reviews and Meta-analysis statement (PRISMA; Moher et al., 2009). Methods of the analysis and inclusion criteria were also registered in Prospero (protocol #CRD42021247635, register: Y.-L. Jao). The following search terms were used:

*(Dementia OR Alzheimer) AND (behavioral symptom OR agitation OR wandering OR depression OR depressed OR aggression OR affect OR engagement OR neuropsychiatric symptoms OR apathy OR resistiveness OR Behavioral and Psychological Disorders OR anxiety) AND (bright light OR environmental light OR ambient light OR tailored light OR indirect light OR light therapy OR LED light)*

### Inclusion Criteria and Article Selection

Inclusion criteria were intervention studies evaluating the effect of ambient BL on any BPSD in persons with dementia. In this review, ambient BL is defined as a lighting intervention that (a) functions to change the illuminance in the room, rather than only a specific small area of the room, and (b) allows exposure to the lighting without requiring participants to sit near and look directly at the light device. Review articles were not included but were used for additional eligible articles. Nonexperimental studies, qualitative research, editorials, commentaries, expert opinions, case studies, conference abstracts, and study protocols with no results reported were excluded. Studies that focused on people with mild cognitive impairment were also excluded.

**Figure 1.**
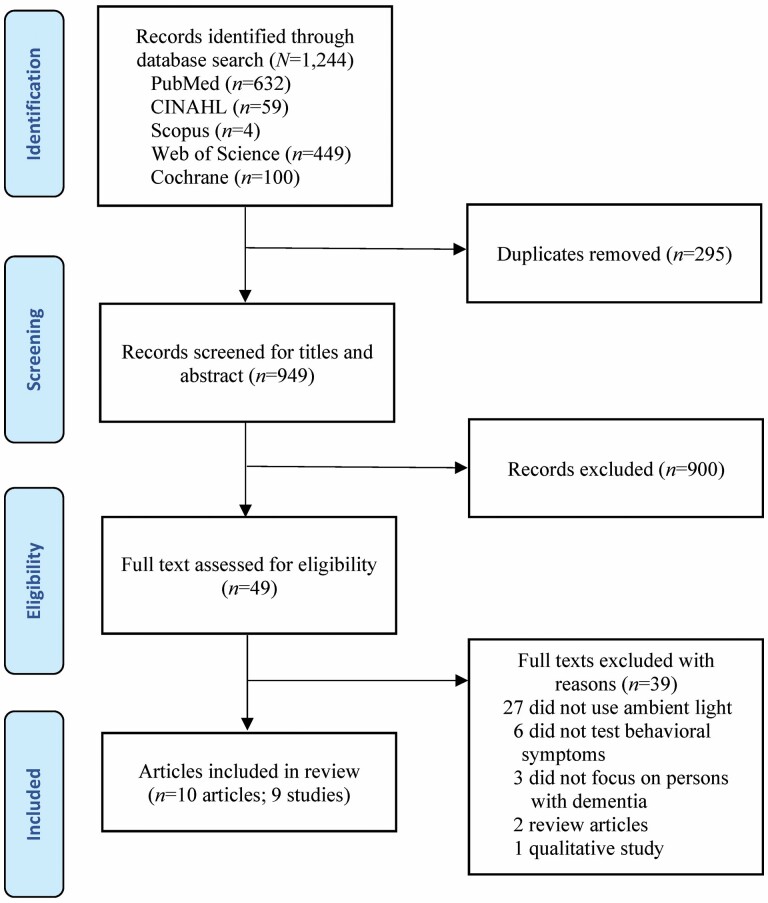
PRISMA diagram.

Article selection was conducted independently by two coauthors (Y.-L. Jao and J. Parajuli), and disagreements were reconciled (Figure 1; Moher et al., 2009). The search yielded 1,244 articles. After eliminating duplicates, 949 articles were screened for titles and abstracts, and 49 potentially eligible articles were identified. Next, two coauthors (Y.-L. Jao and J. Parajuli) independently reviewed the full text and identified 10 eligible articles, nine studies (two articles were from the same study), to be included in the review.

### Data Extraction, Quality Assessment, and Data Synthesis

Data extraction and quality assessment were conducted by three coauthors (Y.-L. Jao, J. Parajuli, and L. McNally). Each article was assigned to two of the three coauthors (Y.-L. Jao, J. Parajuli, and L. McNally) for data extraction and quality assessment to ensure accuracy. Discrepancies were reconciled by consensus (Table 1). The lead author (Y.-L. Jao) and two coauthors (J. Wang and N. Wang) further verified the extracted information, which included the design, sampling, setting, intervention and control, outcome measures, and study results. Information on lighting design included the lighting types, installation areas, parameters, timing, and durations. For lighting parameters, information about target lighting dosage and actual dosage were extracted to evaluate the intervention fidelity of each study.

**Table 1.** Study Characteristics

Study	Design, setting and sample size	Key inclusion and exclusion criteria	Outcomes and Measures	Results
Barrick et al. (2010)	Quasi-experimental study. Cluster-unit cross-over design. Four lighting conditions: (a) morning BL (7–11 a.m.), (b) evening BL (4–8 p.m.), (c) all-day BL (7 a.m.–8 p.m.), (d) all-day DL (7 a.m.–8 p.m.) (Control). *N* = 66 in a psychiatric hospital in one site and a dementia-specific residential care facility in the other site.	Inclusion: Dementia diagnosis, including mild to severe dementia. Exclusion: Bipolar disorder, diabetic retinopathy, moderate or severe macular degeneration, and absence of a lens Note: Not limited to individuals with agitation or depressive symptoms at baseline.	Agitation: CMAI Researcher observation.	▪ CMAI: Compared to the DL condition (control), resident CMAI scores were not significantly different in all three BL conditions. ▪ Researcher observation: **◦** Mild–moderate dementia: Compared to DL (control), agitation was higher during morning BL (OR = 1.57, *p* = .003], evening BL (OR = 1.90, *p* ≤ .001), and all-day BL (OR = 1.63, *p* = .001). **◦** Severe dementia: Compared to DL (control), agitation was marginally higher during morning BL (OR = 1.22, *p* = .055) but no significant difference for evening BL or all-day BL.
Hickman et al. (2007)			Depressive symptoms: CSDD.	▪ Depressive symptoms: Men experienced significantly more depressive symptoms in morning BL (2.62 points, *p* = .007) and marginally higher depressive symptoms in all-day BL (1.65, *p* = .08) than DL. Women experienced marginally fewer depressive symptoms in the morning BL than DL (control condition; −1.61, *p* = .09). No significant changes in other light conditions relative to control. ▪ The study reported no intervention-related side effects.
Figueiro et al. (2014)	Quasi-experimental, pretest–posttest design. *N* = 14 in NHs.	Inclusion: Mild to moderate dementia (DSM-IV). Exclusion: Major organ failure, major illness, head injury, uncontrolled generalized disorders, use of psychotropic medications, obstructing cataracts, macular degeneration, and blindness. Notes: Not limited to participants with agitation or depressive symptoms. The sample had a very low level of agitation at baseline.	Depressive symptoms: CSDD Agitation: CMAI	▪ Depressive symptoms: Reduced significantly from baseline to during intervention (*p* = .03; baseline: 12; during intervention: 6; postintervention: 9). ▪ Agitation: Reduced significantly from baseline to during intervention (*p* = .037) and from baseline to postintervention (*p* = .03; baseline: 38.2, during intervention:31.2; postintervention: 32.3). ▪ No side effect reported.
Figueiro et al. (2015)	Quasi-experimental, pretest–posttest design. *N* = 35 in community.	Inclusion: Mild to moderate dementia, CDR: 1–2, MMSE: 12–24. Note: Participants had a low level of depressive symptoms at baseline.	Depressive symptoms: CSDD (caregiver rating) GDS (self-report).	▪ CSDD: Did not reduce significantly from baseline: 7.00; during intervention: 7.00; postintervention: 7.16. ▪ GDS: Reduced significantly from baseline to postintervention (*p* = .005) and approached significance from intervention to postintervention (*p* = .083; baseline: 3.17; during intervention: 2.57; postintervention: 2.18). ▪ Side effects: No side effects reported.
Figueiro et al. (2019)	RCT, placebo-controlled, cross-over design. One week for baseline before each intervention, 4 weeks for active intervention, 4 weeks for control, and 4 weeks for washout. *N* = 46 in four assisted-living and four long-term care facilities.	Inclusion: Dementia diagnosis (DSM-IV), MMSE 4–24 (mild to severe dementia), BIMS 3–12 (moderate to severe cognitive impairment), PSQI > 5 (sleep disturbance). Exclusion: Major organ failure, a major illness, a history of head injury, uncontrolled generalized disorders, obstructing cataracts, macular degeneration, blindness, or used psychotropic medicine, severe sleep apnea, or restless legs syndrome.	Depressive symptoms: CSDD Agitation: CMAI	▪ Depressive symptoms: Reduced significantly from baseline mild depressive symptoms to postintervention no depressive symptoms in the intervention condition (baseline: 10.3; postintervention: 7.05). The control condition did not decrease significantly (baseline: 10.73; postintervention: 9.61). The intervention group had significantly more effective than the control group (*p* = .04). ▪ Agitation: Significant decrease of agitation after intervention condition (baseline: 42.65; postintervention: 37.14) and nonsignificant decrease in the control condition (baseline: 42.71; postintervention: 41.21). The intervention group had significantly more effective than the control group (*p* = .02). ▪ Side effects: No side effects reported.
Figueiro et al. (2020)	Quasi-experimental, pretest–posttest design. *N* = 47 in three assisted-living facilities and six long-term care facilities.	Inclusion: Dementia diagnosis based on DSM-IV, MMSE 4–24 or BIMS 3-12, PSQI > 5.	Depressive symptoms: CSDD Agitation: CMAI	▪ Depressive symptoms: Reduced significantly at weeks 3, 9, 17, and 25 compared to baseline (*p* < .001). Week 25 was also significantly lower than week 3 (*p* < .001; baseline: 11.36, week 3: 7.47, week 9: 6.02, week 17: 5.95, week 25: 4.18). ▪ Agitation: Agitation was significantly lower at weeks 3, 9, 17, and 25 compared to baseline (*p* < .001). Baseline: 47.10, week 3: 40.89, week 9: 37.54, week 17: 36.8, and week 25: 35.33. Agitation decreased more in severe dementia compared to mild–moderate dementia. ▪ Side effects: No side effects reported.
Munch et al. (2017)	Quasi-experimental study, with an intervention group and a control group. Analysis was not based on the intervention or control group but was based on the actual lighting exposure (high-light group > 417.24, *n* = 44; low-light group < 417.24, *n* = 45). *N* = 89 in an NH.	Inclusion: >50 years old, dementia diagnosis (DSM-IV), absence of visual blindness.	Agitation: CMAI Pleasure, general alertness, anger, sadness, and fear: OERS (1–5) for each item.	▪ Agitation: No significant difference. ▪ Pleasure: Significantly higher in the high-light group than the low-light group (low-light: approximately 1.9, high-light: 2.1, *p* = .037). ▪General alertness: Significantly higher in the high-light group (low-light: approximately 4.5, high-light: approximately 4.7, *p* = .004). ▪ Sadness: No significant difference. ▪ Fear: No significant difference. ▪ Side effects: No side effects reported.
Van Hoof, Schoutens, et al. (2009)	RCT, cross-over design, two groups. Both groups received BL 1 and BL 2, just in a different order. *N* = 22 in a psychogeriatric daycare setting.	Inclusion criteria: Dementia diagnosis.	Depressive/sad behavior (0–18), anxious behavior (0–18), apathetic behavior (0–18), restless behavior (0–15), and disturbances of consciousness (0–21): GIP	▪ Depressive/sad behavior: Group 1: Significantly increased from two post BL 1 to four post BL 2 (*p* = .028), Group 2: no significant difference. ▪ Anxious behavior: Group 1: Significantly increased from 0 post BL 1 to 2.5 post BL 2 (*p* = .015). Group 2: No significant difference. ▪ Apathy: No significant difference in neither group. ▪ Restless behavior: No significant difference in either group. ▪ Disturbances of consciousness: Group 2 showed a marginal decrease from 6.5 in BL 1 to 7.5 in BL 2 (*p* = .065) but no difference for Group 1. ▪ Side effects: No side effects reported.
Van Hoof, Aarts, et al. (2009)	Quasi-experimental design with an intervention and a control group. Intervention group: BL 1 × 3 weeks → control × 2 weeks (washout period) → BL 2 × 3 weeks. Control group: DL all day during the study period. *N* = 26 in psychogeriatric ward in the hospital.	Inclusion: Dementia diagnosis. Exclusion: Bed-ridden residents.	Depressive/sad behavior, anxious behavior apathetic behavior, restlessness behavior, and disturbances of consciousness: GIP	▪ Depressive/sad behavior: ◦ Intervention: Decreased from 2.5 at baseline, to 2 after BL 1, and increased to 2.5 after BL 2. ◦ Control: Decreased from 4.5 at baseline, to 2 after BL 1, and 4 after BL 2. ◦ Significance for before and after intervention was not reported. ▪ Anxious behavior: ◦ Intervention: Decreased from 1 at baseline to 0.5 after BL 1, but increased to 2 after BL 2. ◦ Control: 5.5 at baseline, reduced to 3.5 after BL 1, but increased to 8 after BL 2. ◦ Significance for before and after intervention was not reported. ▪ Apathy: ◦ BL1: Increased from 7.5 at baseline to 10 (*p* = .017) after intervention. ◦ BL2: No significant difference. ▪ Restless behavior: ◦ BL1: Reduced from 3.5 at baseline to 2.5 (*p* = .005) after intervention. ◦ BL2: No significant difference. ▪ Disturbance of consciousness: ◦ BL1: No significant difference. ◦ BL2: No significant difference. ▪ Side effects: No side effects reported.
Wahnschaffe et al. (2017)	Quasi-experimental, pretest–posttest design. *N* = 15 in an NH.	Inclusion: Dementia diagnosis based on ICD-10, capable to tolerate physical mobility. Exclusion: Bed-ridden individuals.	Agitation: CMAI	▪ Agitation: Reduced significantly post the intervention (baseline: 30.17; postintervention: 27.92; *p* = .043). ▪ Side effects: No side effects reported.

*Notes*. BIMS = Brief Interview for Mental Status; BL = bright light; CDR = Clinical Dementia Rating; CMAI = Cohen-Mansfield Agitation Inventory; CSDD = Cornell Scale for Depression in Dementia; DL = dim light; DSM-IV = Diagnostic and Statistical Manual of Mental Disorders, fourth edition; ICD 10 = International Statistical Classification of Disease and Related Health Problems 10th edition; GDS = Geriatric Depression Scale; GIP = Dutch Behavior Observation Scale for Intramural Psychogeriatrics; MMSE = Mini-Mental State Examination; NH = nursing home; OR = odds ratio; OERS = Observed Emotion Rating Scale; PSQI = Pittsburg Sleep Quality Index; RCT = randomized controlled trial.

Article quality assessment was conducted using the Johns Hopkins Nursing Evidence-Based Practice Evidence level and Quality Guide (Newhouse et al., 2005) and the Cochrane Collaboration’s Tool for Assessment of Risk of Bias in Randomized Trials (Higgins et al., 2011). The lead author (Y.-L. Jao) verified the quality assessment.

Using the Johns Hopkins tools, the evidence level is rated on a 3-point scale, based on study design (Level 1: randomized control trial [RCT], Level 2: quasi-experimental study, and Level 3: nonexperimental study; Newhouse et al., 2005). For the quality, each article was categorized into one of three levels: (a) high, (b) good, and (c) low/major flaws (Newhouse et al., 2005). The risk of bias was assessed based on seven bias domains rated as low, unclear, or high risk of bias (Higgins et al., 2011).

## Results

### Study Characteristics

#### Study design

Nine studies were included in this review, of which two articles reported results from the same study (Barrick et al., 2010; Hickman et al., 2007), resulting in ten articles in total. Most studies were quasi-experimental with only one RCT (Figueiro et al., 2019). Among the eight quasi-experimental studies, five used a single group, pretest–posttest design (Barrick et al., 2010; Figueiro et al., 2014, 2015, 2020; Hickman et al., 2007; Wahnschaffe et al., 2017) and three involved an intervention group and a control group but were not randomized to condition (Münch et al., 2017; Van Hoof, Aarts, et al., 2009; Van Hoof, Schoutens, et al., 2009; Table 1).

#### Setting

Five studies were conducted in long-term care settings, three in nursing homes (NHs; Figueiro et al., 2014; Münch et al., 2017; Wahnschaffe et al., 2017), and two in assisted living and other long-term care communities (Figueiro et al., 2019, 2020). Among the other four studies, one study recruited participants from community (Figueiro et al., 2015), one from psychogeriatric daycare (Van Hoof, Schoutens, et al., 2009), one from a hospital psychogeriatric unit (Van Hoof, Aarts, et al., 2009), and one study recruited participants from one hospital and one NH facility (Barrick et al., 2010; Hickman et al., 2007).

#### Sample

Study sample size ranged from 14 (Figueiro et al., 2014) to 89 participants (Münch et al., 2017). All studies used the diagnosis of dementia as an inclusion criterion. Two studies only included participants with mild to moderate dementia (Figueiro et al., 2014, 2015), other studies did not limit to specific stages of dementia. While all studies evaluated the effect of lighting interventions on BPSD, none of the studies selected for individuals with the BPSD of interest at baseline.

#### Study quality

Only one study was an RCT (Level 1 evidence) and had a relatively low risk of bias (Figueiro et al., 2019). All others were quasi-experimental studies (Level 2 evidence) and had a high risk of bias. Five studies were of good quality (Barrick et al., 2010; Figuerio et al., 2015, 2019, 2020; Hickman et al., 2007; Munch et al., 2017), and four were of low quality (Table 2).

**Table 2.** Level of Evidence and Quality Evaluation

Study	Level of evidence	Level of quality	Risk of bias						
			Random sequence generation	Allocation concealment	Blinding participants and researchers	Blinding of outcome assessment	Incomplete outcome data	Selective reporting	Other bias
Barrick et al. (2010)	Level II	Good	N/A	N/A	H	H	L	L	H
Hickman et al. (2007)	Level II	Good	N/A	N/A	H	H	H	L	H
Figueiro et al. (2014)	Level II	Low	N/A	N/A	H	H	L	L	L
Figueiro et al. (2015)	Level II	Good	N/A	N/A	H	H	L	L	L
Figueiro et al. (2019)	Level I	Good	L	L	H	H	L	L	H
Figueiro et al. (2020)	Level II	Good	N/A	N/A	H	H	H	L	H
Munch et al. (2017)	Level II	Low	N/A	N/A	H	L	L	L	H
Van Hoof, Schoutens, et al. (2009)	Level II	Low	N/A	N/A	H	H	H	L	H
Van Hoof, Aarts, et al. (2009)	Level II	Low	N/A	N/A	H	H	L	L	H
Wahnschaffe et al. (2017)	Level II	Low	N/A	N/A	H	H	H	L	H

*Note:* H = high risk of bias; L = low risk of bias; N/A = not applicable.

### Intervention Design

#### Lighting types and installations

Five of the nine studies used a ceiling light for their ambient lighting interventions. Of the other studies, two used floor lamps oriented upward toward the ceiling (Figueiro et al., 2014, 2015), and two used a hybrid lighting configuration consisting of large light tables, lightboxes, and floor lamps (Figueiro et al., 2019, 2020). Six studies used fluorescent lights, one used LED lights (Figueiro et al., 2019), and two did not specify the type of lights used (Barrick et al., 2010; Figueiro et al., 2020; Hickman et al., 2007). For the location of lighting installation, one study installed lights in participants’ bedrooms (Figueiro et al., 2014), one in both bedrooms and common areas (Figueiro et al., 2019), and the rest of the six studies exclusively in common areas, such as living, activity, and dining rooms (Table 3).

**Table 3.** Parameters of Bright Light Intervention Design Across Studies

Study	Lighting device	Lighting area	Illuminance (Lux)	CCT (K)	Circadian Stimulus	BL hours/day (hr/day)	Nighttime light	Duration
Barrick et al. (2010)	Ceiling light	Common area(s)	Others: *Intervention:* Target: 2,000–3,000 Actual: 2,535 at one site and 2,638 in the other site. *Control:* Target: 500–600 Actual: 617 at one site and 591 at the other site.	NS		*Intervention* Three BL conditions: (1) morning BL Target: 4 (7–11 a.m.) Actual: Approximately 3 (2) evening BL Target: 4 (4–8 p.m.) Actual: Approximately 3 (3) all-day BL Target: 13 (7 a.m.–8 p.m.) Actual: Approximately 7–8 *Control* (4) all-day DL (7 a.m.–8 p.m.) Actual: NS		Three weeks per intervention period, the periods repeated. Twenty-two intervention periods at one site and eight at the other. Average exposure for each participant: 4.2 intervention periods in hospital and 7.4 periods in residential care.
Hickman et al. (2007)								
Figueiro et al. (2014)	Fluorescent lamps upward to the ceiling	Bedroom	Eye level: *Baseline* Actual: Room: 66 ± 130 lux (V) Individual sensor: 103 ± 31 circadian light (V) *Intervention* Actual: Room: 324 ± 190 lux (V) Individual sensor: 373 ± 121 circadian light (V)	9,325 K	*Baseline* Actual (individual sensor): CS = 0.06 ± 0.01 at wrist *Intervention* Target: 0.375 Actual (individual sensor): CS = 0.1 ± 0.01 at wrist	10–12 (wake time from 6–8 a.m. to 6 p.m.)		4 weeks
Figueiro et al. (2015)	Fluorescent lamps upward to the ceiling	Common area(s)	Eye level: *Baseline*: NS *Intervention*: Target: ≥350–400 (V)	*Baseline:* NS *Intervention*: 9,325 K	*Baseline* Actual: 0.11 ± 0.01 *Intervention* Target: 0.375 Actual: 0.15 ± 0.01 *Postintervention* Actual: 0.09 ± 0.01 Measured via individual sensors at chest height.	10–12 (wake time to 6 p.m.)		4 weeks
Figueiro et al. (2019)	LED lamps, light boxes, and LED light tables	Bedroom and common area(s)	Eye level: *Baseline* NS *Intervention* Lamps: 550 or 600 (V) Light boxes: 350 (V) Light table: 750 (V) *Control*: Lamps: 110 Light boxes: 100 Light table: 200	*Baseline:* NS *Intervention* Lamps: 7,000 or 5,000 K Light boxes: 6,000 K Light table: 5,000 K *Control* Lamps: 2,000 or 2,700 K Light boxes: 2,700 K Light table: 2,700 K	*Baseline:* NS *Intervention* Target: 0.4 Actual: ≈0.17 at chest *Control:* Target: CS < 0.1	10–12 (wake time from 6–8 a.m. to 6 p.m.)	CS < 0.1 (6 p.m. to wake time)	4 weeks
Figueiro et al. (2020)	Custom-built lamps, light boxes, and light tables	Bedroom and common area(s)	Eye level: NS	NS	*Baseline*: NS *Intervention*: Target: 0.4 at eye level	10–12 (wake time from 6–8 a.m. to 6 p.m.)	CS < 0.1, 6 p.m.–6 a.m. to 8 a.m.	6 months
Munch et al. (2017)	Fluorescent ceiling lights	Common area(s)	Eye level: *Baseline*: NS *Intervention*: Target: ≈1,000 (V) *Control*: NS *Actual*: no significant difference between intervention and control	*Intervention* 6,500 K (cold-white) and 2,700 K (warm-white) *Control* 2,700 K (warm-white)		7 (9 a.m. to 4 p.m.)	Controlled but not clearly specified	8 weeks
Van Hoof, Schoutens, et al. (2009)	Fluorescent ceiling light	Common area(s)	Eye level: *Baseline*: 50 lux (H). *Intervention*: BL 1: Target: 500 lux (H) Actual: 427–469 lux (V) BL 2: Target: 500 lux (H) Actual: 375–433 lux (V)	*Baseline:* NS *Intervention* BL 1: Target: 2,700 K Actual: *M* = 2,823–3,461 K BL 2: *M* = Target: 17,000 K Actual: *M* = 7,364–8,358 K		10 (8 a.m. to 6 p.m. daily during both interventions)		Approximately 2.5 weeks for BL 1 and 4.5 weeks for BL 2, 7 weeks in total
Van Hoof, Aarts, et al. (2009)	Fluorescent ceiling light	Common area(s)	Eye level: *Baseline*: Actual: Intervention group: 86 lux (V) Control group: 156 lux (V) *Intervention*: Target: 1,000 lux (V) Actual: BL 1: 413 lux (V) BL 2: 410 lux (V) *Control* Actual: BL 1: 144 (V) BL 2: 43 (V) All actual lighting was measured throughout the day, including daytime and nighttime *Others*: Baseline: NS Intervention: Ceiling light: 1,750– 1,810 lux (yellowish). (H) at table height. Control: NS	*Baseline:* 2,700 K *Intervention* Ceiling light: ▪BL 1: 6,500 K (bluish) ▪BL 2: 2,700 K (yellowish) (H) at table height. *Control:* Target: 2,700 K		11 (8 a.m. to 6 p.m.)		Three weeks per intervention for 6 weeks of intervention in total with a 2-week washout period in between
Wahnschaffe et al. (2017)	Fluorescent ceiling light	Common area(s)	Eye level: *Baseline*: NS *Intervention*: Actual: 389 (V)	*Baseline:* NS *Intervention* Actual: BL 4,440 K (bluish); NL 1,747 K		11 (BL: 10 a.m. to 3 p.m., NL: 8 p.m. to 5 a.m.)	*Baseline* NA *Intervention* Actual: 34 lux (V)	13 days

*Notes:* BL = bright light; CCT = corrected color temperature; CS = circadian stimulus; DL = dim light; H = horizontal illuminance; NL = night light; NS = not specified; V = vertical illuminance.

#### Lighting parameters

Lighting parameters selected and maintained for lighting interventions are related to lighting condition intensity and color. Intensity may be controlled by the designed photopic illuminance levels at the selected levels, planes, or heights, while the corrected color temperature (CCT) of light sources modulates the lighting color conditions. In addition to intensity and color, circadian stimulus (CS) has been recently adopted as a metric to reflect the effectiveness of the spectrally weighted density of light incident at the eye from no melatonin suppression (CS = 0.1) to saturation (CS = 0.7; Rea & Figueiro, 2018). The CS calculation considers both the lighting intensity and lighting spectra. The details of these three lighting parameters (illuminance, CCT, and CS) in the selected studies are discussed in the following sections.

##### Illuminance (lux)

.—All studies measured the photopic illuminance in their lighting interventions, except that one study did not report a measure of illuminance (Figueiro et al., 2020). The majority of the studies measured photopic illuminance at the eye level, one measured at the table height (Van Hoof, Aarts, et al., 2009), and one study did not specify (Barrick et al., 2010; Hickman et al., 2007). Most of the studies aimed to maintain BL at the eye level during the daytime, but the targeted illuminance varied across studies, ranging from 300 lux (Figueiro et al., 2014) to 1,000 lux (Münch et al., 2017; Van Hoof, Aarts, et al., 2009). One study (Barrick et al., 2010; Hickman et al., 2007) appeared to maintain much higher illuminance (2,000–3,000 lux), but the illuminance was not measured at eye level. In addition to target illuminance, four studies also measured actual illuminance in the room or received by the participants (Barrick et al., 2010; Hickman et al., 2007; Münch et al., 2017; Van Hoof, Aarts, et al., 2009; Van Hoof, Schoutens, et al., 2009).

##### Correlated color temperature (K)

.—With the exception of two studies (Barrick et al., 2010; Figueiro et al., 2020; Hickman et al., 2007), all other studies controlled lighting color via CCT. Among studies that maintained a consistent CCT, it widely varied from 2,700 K to 17,000 K (Van Hoof, Schoutens, et al., 2009); one at 4,440 K (Wahnschaffe et al., 2017), two at 6,500 K (Münch et al., 2017; Van Hoof, Aarts, et al., 2009), one at 5,000–7,000 K (Figueiro et al., 2019), two at 9,325 K (Figueiro et al., 2014, 2015), and one with two conditions―2,700 K and 17,000 K (Van Hoof, Schoutens, et al., 2009). Notably, one study measured the actual CCT during the intervention period and reported very different levels than those targeted (Van Hoof, Schoutens, et al., 2009).

##### Circadian stimulus

.—CS level quantifies circadian light (Rea & Figueiro, 2018) by lighting intensity and color information (Rea & Figueiro, 2018). Intervention design incorporated CS in four studies (Figueiro et al., 2014, 2015, 2019, 2020). Daysimeter, a small device that continuously measures participant CS levels, assessed the target CS of 0.375–0.4 during the day. Two studies further controlled light during the night with CS < 0.1 (Figueiro et al., 2019, 2020). Notably, two studies measured the actual CS during the intervention period and reported much lower CS levels (0.1–0.15) than the targeted (0.375–0.4; Figueiro et al., 2014, 2015).

Overall, the lighting parameters and measurement methods varied widely across studies. All but two studies (Figueiro et al., 2020; Wahnschaffe et al., 2017) reported both the target and actual lighting dosage. Among the seven studies that reported both target and actual lighting dosages, only one study, which used a wide range of 2,000–3,000 lux as the target, reported that the actual lighting dosages were within the target range (Barrick et al., 2010; Hickman et al., 2007). Most other studies used a more specific target dosage and reported moderate to major discrepancies between the target and actual lighting for all lighting parameters. For example, in a study by Munch et al. (2017), the lighting was targeted at approximately 1,000 lux for the intervention group and 310 lux for the control group; yet the actual lighting exposure in the two groups was not significantly different (Munch et al., 2017). In another study, the lighting was targeted at 1,000 lux or higher provided via ceiling lights at 1,750–1,810 lux, but the actual lighting dosage was 410–413 lux on average (Van Hoof, Aarts, et al., 2009). However, in this study, it is unclear whether the measurements for actual lighting dosage were all taken during BL hours (Van Hoof, Aarts, et al., 2009). In another study, one lighting condition was targeted at CCT = 17,000 K, yet the actual lighting dosage on average was only CCT = 7,364 K in one group and CCT = 8,358 K in the other group (Van Hoof, Schoutens, et al., 2009). In the three studies that focused on CS as the parameter, while the intervention was targeted at CS = 0.375 or 0.4, the actual lighting dosage on average was CS = 0.10–0.17 (Figueiro et al., 2014, 2015, 2019).

#### Time control

Time control widely varied across studies. Regarding timing and duration, one study tested the effect of lighting intervention during three different daytime periods (Barrick et al., 2010; Hickman et al., 2007), while the other eight studies adopted a continuous BL exposure ranging from 7 to 12 hr during the daytime. Also, four studies incorporated the nighttime schedule, with dim and/or warm light, using 66 lux (Figueiro et al., 2014), CS < 0.1(Figueiro et al., 2019), 34 lux (Wahnschaffe et al., 2017), and one study did not specify the details (Münch et al., 2017). The duration of the intervention varied from 2 weeks (Wahnschaffe et al., 2017) to 6 months (Figueiro et al., 2020), with 4 weeks being the most common period.

### Study Findings Outcomes

Depressive symptoms were the most tested BPSD, followed by agitation. Other BPSD tested included apathy, disturbances of consciousness, restlessness behavior, anxiety, pleasure, general alertness, anger, sadness, and fear. The effect of ambient BL on each BPSD is described later.

#### Depressive symptoms

Seven studies evaluated the effect of ambient BL on depressive symptoms (Hickman et al., 2007; Figueiro et al., 2014, 2015, 2019, 2020; Van Hoof, Aarts, et al., 2009; Van Hoof, Schoutens, et al., 2009). Three studies reported significant effects in reducing depressive symptoms (Figueiro et al., 2014, 2019, 2020), and one study reported a significant effect (*p* = .005) based on self-report, but no significant difference based on caregiver-report Cornell Scale for Depression in Dementia (CSDD; Figueiro et al., 2015). The lighting interventions in the first three studies were bluish BL with high CS level (300–750 lux; 5,000–9,325 K; target CS = 0.375–0.4; actual CS = 0.1–0.17; Figueiro et al., 2014, 2015, 2019). The BL in the last study was targeted at CS = 0.4 (Figueiro et al., 2020). All four studies provided BL for 10–12 hr/day and three of the studies also maintained dim light during the nighttime at 66 lux (Figueiro et al., 2014) or CS < 0.1 (Figueiro et al., 2019, 2020).

For the other three studies, two reported mixed results (Hickman et al., 2007; Van Hoof, Schoutens, et al., 2009), and one study showed no significant difference in depressive symptoms (Van Hoof, Aarts, et al., 2009). Specifically, Hickman et al. (2007) evaluated a BL intervention (2,000–3,000 lux) in three different exposure periods (morning, afternoon, and all day) as compared to dim light (500–600 lux). Male participants showed a significantly higher level of depressive symptoms in morning BL (increased by 2.62 on CSDD, *p* = .007) and higher depressive symptoms with marginal significance in all-day BL (increased by 1.65 on CSDD, *p* = .08). In contrast, female participants showed marginally lower depressive symptoms (deceased by 1.61, *p* = .09) in the morning BL compared to the dim light but no significant difference on depressive symptoms in other BL conditions. This study did not specify the CCT or the measurement method for the lighting intensity, making it challenging to synthesize the results with other studies (Hickman et al., 2007).

Van Hoof, Schoutens, et al. (2009) applied a designed lighting intensity (500 lux) along with very high CCT (17,000 K) versus low CCT (2,700 K) at vertical eye level. The study reported that participants’ depressive symptoms showed a slight but statistically significant increase from low CCT to high CCT in one group but no significant change in the other group. Notably, the two groups of participants received slightly different light intensity levels; the group with lower intensity and lower CCT (375 lux, 7,346 K) showed decreases in depressive symptoms, while the high-CCT group (433 lux, 8,358 K) showed no difference. Also, the participants in the high CCT lighting scenario received relatively lower mean illuminance levels in general, relative to the low CCT lighting scenario, which complicates the interpretation of the effects of lighting intensity. The other study by Van Hoof, Aarts, et al. (2009) tested the effect of high-intensity BL (target level ≥1,000 lux) with a higher CCT (6,500 K) and a lower CCT (2,700 K) as compared to the dim lighting (target level 200 lux, 2,700 K). Depressive symptoms were very similar across the three lighting conditions. Significance levels were not reported. Among the three studies that did not show positive results, none controlled nighttime lighting conditions.

#### Agitation

Six studies examined the effect of ambient BL on agitation (Barrick et al., 2010; Figueiro et al., 2014, 2019, 2020; Münch et al., 2017; Wahnschaffe et al., 2017). Four reported significantly reduced agitation after the intervention, one reported no significant difference (Münch et al., 2017), and another found worsened agitation in the BL groups (Barrick et al., 2010).

The lighting conditions that showed positive results included lighting approximately targeted with high illuminance (350–750 lux at vertical eye level), bluish color temperature (4,440–9,325 K), and high CS (CS = 0.375–0.4), and the intervention was provided 5–12 hr/day for 2 weeks to 6 months (Figueiro et al., 2014, 2019, 2020; Wahnschaffe et al., 2017). Among the four studies, three studies by Figueiro et al. designed the lighting primarily based on CS. While the target CS was 0.375 to 0.4, the actual CS levels received by participants were 0.1 at the wrist (Figueiro et al., 2014) or 0.17 at the chest (Figueiro et al., 2014).

Agitation was reduced from 2.0 (Wahnschaffe et al., 2017) to 12 (Figueiro et al., 2020) points on the Cohen-Mansfield Agitation Inventory (CMAI; rating range = 29–207) (Cohen-Mansfield et al., 1989). Lighting interventions that maintained high CS levels showed the greatest improvement; 5.5–6 points on CMAI after 4 weeks of BL (Figueiro et al., 2014, 2019, 2020) and 10–12 points after 2–6 months of BL (Figueiro et al., 2020). The longitudinal study further reported that the intervention effect was highest for individuals with severe dementia (Figueiro et al., 2020).

The study reporting nonsignificant results maintained cold-white BL (1,000 lux, 6,500 K) during the day, and warm-white dim light (2,700 K) during the night, compared to constant dim warm-white light in the control group (310 lux, 2,700 K; Münch et al., 2017). The study reported fidelity issues resulting in no difference in the actual lighting received between the intervention and control groups. For this reason, the study did not compare the intervention effect as designed; rather, in the analysis, all participants were categorized into high-light (>417 lux) or low-light (<417 lux) group.

The study reporting negative results targeted BL at 2,000–3,000 lux in three different time exposures (morning, afternoon, and all day) compared to dim light (500–600 lux) for 13 hr (Barrick et al., 2010). Participants’ CMAI ratings were not significantly different after any BL condition compared to the dim light. However, results measured via researcher observation showed that participants’ agitation levels were significantly more likely to deteriorate in all three BL conditions as compared to the dim light. Notably, while the BL in this study seemed to use a much higher illuminance level, the study did not specify lighting color quantities or the measurement placements, making it challenging to compare the lighting scheme with other studies (Barrick et al., 2010).

#### Other BPSD

Münch et al. (2017) evaluated the effect of lighting interventions on positive and negative affect in 89 residents in NHs. As mentioned previously, this study had intervention fidelity issues. The results revealed that residents in the high-light group (>417 lux) showed significantly higher levels of pleasure and general alertness than the low-light group (<417 lux). The average pleasure level was significantly higher in the high-light group than in the low-light group. Similarly, general alertness in the high-light group was slightly higher in the high-light group compared to the low-light group with a statistical significance. No significant difference was reported on sadness and fear.

Van Hoof, Aarts, et al. (2009) and Van Hoof, Schoutens, et al. (2009) tested the effect of lighting interventions in two studies. These studies compared the effect of two lighting interventions with the same lighting intensity, but different lighting color temperatures, more yellowish light (lower CCT) versus more bluish light (higher CCT). The first targeted BL 500 lux with high CCT (17,000 K) as compared to low CCT (2,700 K; Van Hoof, Schoutens, et al., 2009), and the second study tested two BL interventions both targeted at 1,000 lux at table height and one with high CCT (6,500 K) and the other with low CCT (2,700 K) as compared to control group with dim light (200 lux, 2,700 K; Van Hoof, Aarts, et al., 2009). These two studies examined anxious behaviors, apathy, restlessness behavior, and disturbances of consciousness.

The effect of ambient BL on anxious behaviors was mixed. The first study revealed that participants showed worsening anxious behavior after being exposed to high-CCT BL as compared to low-CCT BL. (Van Hoof, Schoutens, et al., 2009). However, this significantly negative result was only found in participants in one of the intervention groups but not the other group, who were exposed to slightly different lighting. In the second study, participants’ anxious behavior was decreased slightly after being exposed to BL with high CCT but was increased after the BL with low CCT (Van Hoof, Aarts, et al., 2009). The significance level for this outcome variable was not reported.

The effect of ambient BL on apathy was not consistent across the two studies. In the first study, participants’ apathy showed no difference after exposure to a relatively low-level ambient light, either with high CCT or low CCT (Van Hoof, Schoutens, et al., 2009). In the second study (Van Hoof, Aarts, et al., 2009), participants’ apathy levels showed no change after being exposed to BL with high-intensity and low CCT. However, apathy level significantly increased after being exposed to BL with the same intensity but high CCT (6,500 K).

For restless behavior, results were mixed. In the first study, restless behavior showed no difference after BL with high CCT or low CCT (Van Hoof, Schoutens, et al., 2009). In the second study (Van Hoof, Aarts, et al., 2009), restlessness was not significantly changed after exposure to BL with high-intensity and low CCT. However, in the same group of participants’, restless behaviors showed a slight but statically significant decrease after exposure to the BL with the same intensity but higher CCT. In terms of disturbance of consciousness, neither study showed any significant differences in disturbance of consciousness (Van Hoof, Aarts, et al., 2009; Van Hoof, Schoutens, et al., 2009).

#### Adverse events

Among the nine studies reviewed, none reported adverse events associated with the intervention. One study specifically found no intervention-related side effects (Hickman et al., 2007), while the other studies did not mention any adverse side effects.

## Discussion

### Effect of Ambient BL

#### Depressive symptoms

Overall, evidence from seven studies showed mixed results on the effect of BL on depressive symptoms. In the four studies conducted by Figueiro et al., BL significantly reduced depressive symptoms for persons living with dementia (Figueiro et al., 2014, 2015, 2019, 2020). Specifically, providing bluish BL approximately targeted at 350–750 lux, 5,000–9,325 K, and high CS (target at CS = 0.375–0.4 and actual at CS = 0.1–0.17), 10–12 hr/day for at least 4 weeks significantly reduced depressive symptoms in persons with dementia at mild to severe stages across the community and long-term care settings. Furthermore, in a longitudinal study, the BL effect increased when continuing the lighting after 4 weeks for a 6-month period (Figueiro et al., 2020).

However, among the other three studies, two showed a deterioration in depressive symptoms (Hickman et al., 2007; Van Hoof, Schoutens, et al., 2009), and one showed no changes in depressive symptoms (Van Hoof, Aarts, et al., 2009). The study by Hickman et al. (2007) reported opposite results between genders with increased depressive symptoms in males and decreased depressive symptoms in females. Notably, this study used much brighter BL (2,000–3,000 lux) than the other studies and did not specify the color temperature of the BL or their lighting measurement methods, making it challenging to directly compare the BL with other studies. One possible explanation of the mixed results is that the BL in Figueiro’s studies maintained a high CS level while the other studies did not consider the CS levels. Also, the studies that showed a positive effect maintained BL for 4 weeks or longer, while the studies that did not show positive effects only maintained each BL for 3 weeks.

#### Agitation

Some evidence supports the benefit of ambient BL on reducing agitation in persons with dementia; however, the effect of BL may depend on the characteristics of the lighting interventions. Based on evidence from three studies, BL that showed positive results were moderate-intensity bluish BL approximately at 350–750 lux, 4,440–9,325 K for at least 5 hr during the day for at least 2 weeks, and the BL could significantly reduce agitation by 2.3–6.0 points on CMAI scores for persons with dementia across mild to severe stages (Figueiro et al., 2014, 2019; Wahnschaffe et al., 2017). Evidence also showed that the effect seemed stronger when the BL provided a high CS (CS = 0.375–0.4) and was maintained for 10–12 hr/day for 4 weeks, and the effect became 5.5–6 points of reduction on CMAI (Figueiro et al., 2014, 2019, 2020). Notably, participants in these three studies had low baseline agitation levels, which could limit the extent of the intervention effect. Also, all the results were conducted in long-term care facilities. It would be helpful to evaluate the interventions in persons with dementia with agitation across different care settings.

On the other hand, the two studies where BL did not show a positive effect seemed to have some issues with lighting measures and implementations (Barrick et al., 2010; Münch et al., 2017). Importantly, the BL tested in these two studies was very different from the other three studies and had major limitations. In the study by Münch et al. (2017), the actual BL provided had a much lower intensity than the original intervention plan, and the BL intensity varied widely across participants. With that limitation, the study did not compare the effect between the intervention and control groups and only reported results based on the participants’ actual light exposure categorized into the high-light or low-light groups. This analysis approach may largely explain the nonsignificant results. On the other hand, in the study by Barrick et al. (2010), the BL was much brighter (2,000–3,000 lux) than the other studies (350–1,000 lux), and the authors did not specify the color temperature of the BL or specify the measurement methods. Additionally, the participants did not have to have agitation, and their baseline agitation level was not reported. It is unclear whether participants had sufficient agitation to test the intervention effect. These two studies pointed out that not all BL has a positive effect on agitation. Yet, with the major limitations, the results from these two studies need to be interpreted with caution and examined in future research.

#### Other BPSD

The effect of BL on other BPSD was only evaluated in three studies (Munch et al., 2017; Van Hoof, Aarts, et al., 2009; Van Hoof, Schoutens, et al., 2009). In one study, Munch et al. (2017) tested the effect of BL on positive and negative affect and revealed that BL (>417 lux) showed statistically significant improvement on pleasure and general alertness as compared to lower-level light (<417 lux); yet the effect was small and may not be clinically significant. The BL did not significantly change negative affect, including anger and sadness. As mentioned previously, this study had some intervention fidelity issues, such as the actual lighting the participants received was very different from their intended design. The study results may need to be replicated in future research. Especially, the effect of BL on negative affect cannot be excluded and should be evaluated in future research. The other two studies from the same research team (Van Hoof, Aarts, et al., 2009; Van Hoof, Schoutens, et al., 2009) evaluated the effect of BL on anxiety, apathy, restless behaviors, and disturbance of consciousness, and the evidence from both studies do not support the BL effect on these behavioral symptoms. In some cases, participants’ BPSD deteriorated. For example, when the BL was at relatively lower illuminance (375 lux) and high CCT (7,364 K), participants’ depressive symptoms and anxiety significantly increased (Van Hoof, Schoutens, et al., 2009).

### Measurement Approaches of BL

Although most high-illuminance light interventions were performed, the measurement positions were not consistent across the studies. From the visual impact perspective, measuring the received photopic lux at the cornea or the vertical eye level may present a more accurate quantity of light to illustrate the BL exposure, which may maintain the targeted lighting intervention levels (Spitschan et al., 2019). As mentioned earlier, the results reported from the studies that adopted eye-level lighting controls tended to be positive and consistent on agitation improvements. On the other hand, if the illuminance quantities are measured at the horizontal table height or floor level, they may over- or underestimate the BL exposure, which approach which was used in two studies (Barrick et al., 2010; Hickman et al., 2007, Van Hoof, Aarts, et al., 2009). These inconsistent measurement placements might be the reason causing the mixed results on the behavioral symptoms.

Furthermore, most studies showed intervention fidelity issues with actual lighting dosages either not clearly reported or much lower than the target dosage. Two studies only reported either the target lighting or actual lighting levels (Figueiro et al., 2020; Wahnschaffe et al., 2017). Among the studies that reported both target and actual lighting, most studies only reported actual lighting on one parameter, not all parameters (e.g., lux, CCT, and CS), and some studies had measurement issues. For example, the study by Van Hoof, Aarts, et al. (2009) measured actual lighting illuminance multiple times throughout the day, including daytime and nighttime, but it was not clear whether all the measurements were taken only during the BL hours, making it challenging to determine the actual light levels specifically during intervention hours. Lighting distribution varied across different spaces and positions. Also, the changeable outdoor lighting conditions transmitted from various window systems may significantly affect the received lighting conditions, especially for the bedrooms that are typically installed with large windows and abundant daylight availability. Therefore, as situations indicated earlier, the designed lighting levels might not reflect the actual lighting conditions that the participants were exposed to, let alone the accuracy of lighting data, which makes fair comparison among the studies very challenging. Thus, intervention fidelity needs to be carefully addressed when planning and implementing the BL. To accurately assess intervention fidelity, it is important that all lighting intervention studies comprehensively measure and report both the target dosage and the actual lighting dosage received by individual participants during the BL intervention hours. With the rapid development of lighting sensors and monitors, some studies used wearable sensors that enable continuous lighting data collection (Figueiro et al., 2014, 2015, 2019), which tends to be more accurate and complete to represent the realistic lighting exposure conditions. Additionally, the sunlight effects were intentionally removed by closing the window shades in these four studies due to the CS control (Figueiro et al., 2014, 2015, 2019, 2020). In brief, to obtain reliable and accurate lighting condition data, lighting measurement approaches should be carefully selected. We recommend continuous data collection at the eye level of participants while maintaining real-life environmental conditions (e.g., with access to window daylight) and routine living patterns and styles.

### Metrics Used for Lighting Measurement

Photopic illuminance and CCT are the most widely used parameters to quantify the parameter of lighting interventions. However, these metrics may not accurately or comprehensively characterize the spectral irradiance profiles or distributions, especially for the blue light portion. Researchers have shown in humans that the blue portion of light may exert more powerful effects than other spectra on influencing hormone secretion, heart rate, alertness, sleep propensity, and body temperature (Holzman, 2010). The current lighting design in the health care field aims to strengthen the circadian timing system by increasing the blue portion of light during daytime and by reducing the same blue portion of light during the evening hours and the night (Rea & Figueiro, 2018). Therefore, although most studies reported the CCT values used in their BL, the actual blue light intensity is unclear, which may cause different results. The CS metric was particularly developed from the lighting research discipline to address the earlier-described unclear issues about the blue light portion. Four studies (Figueiro et al., 2014, 2015, 2019, 2020) that were mainly led by lighting scientists used this metric and presented positive results on relieving depressive symptoms and agitation. Therefore, future studies might incorporate the CS metric and even more complete radiometric quantities (i.e., spectral power distribution) into the lighting measurements.

On the other hand, from the perspective of practical implementation in clinical practice, the photometric metrics and CCT remain clinically feasible and useful measures for clinical practice for two reasons. First, measuring CS values and spectral power quantities are mostly involved in research-grade lighting measure equipment. Comparatively, regarding the photometric measures and CCT, various lighting sensors are available in the market and are affordable for continuously monitoring lighting conditions for the long term. For practical purposes, it is more important to maintain the designed photopic lux and CCT rather than monitoring the detailed variations of CS and spectra of light. Second, some studies work on the conversion methods or estimation models to extract the key information, such as circadian light and/or blue light intensities from these conventional and widely accepted metrics (Rea & Figueiro, 2018). That makes the approximate assessment of CS conditions possible without a need for CS and radiometric-based measurements. However, other information, such as light source specifications, daylight conditions, and visual properties of interior spaces, are needed for such conversions.

### Nighttime Control in Lighting Schemes

It is also worth noting that several studies (Figueiro et al., 2014, 2019, 2020; Münch et al., 2017; Wahnschaffe et al., 2017) incorporated the nighttime schedule with dim (low lux/CS) and/or warm (low CCT) light into the lighting schemes for the intervention group, and no major negative effects were observed in these studies. Meanwhile, the daytime BL exposure in these four studies also consistently led to positive effects on depressive symptoms, agitation, and others. Setting aside the BL interventions, studies have demonstrated that low-intensity and blue-depleted light may have positive effects on nighttime sleepiness (American Medical Association, 2016; Wahl et al., 2019), which may further affect depressive symptoms, anxiety, and other mood disorders during the daytime. Therefore, nighttime lighting control should be considered for designing the daytime BL intervention.

### Implications for Practice

This review reveals that not all ambient BL showed a positive effect on BPSD; however, when properly designed, ambient BL could have a promising effect on BPSD in persons with dementia, especially on depressive symptoms and agitation. Ambient BL is a noninvasive, not labor-intensive intervention and does not have an adverse effect on persons with dementia, so it can be considered to be installed as a nonpharmacological intervention for persons living with dementia. The ambient BL can be implemented for persons with dementia across stages and across care settings. The ambient lighting can be achieved via ceiling lighting, floor lamps, light tables, or a combination of them. The lighting parameters that mostly showed positive impact are targeted at 350–750 lux vertical at eye level, CCT 4,500–9,325 K, CS = 0.375–0.4 for 10–12 hr during daytime for at least 4 weeks. When installing ambient BL, it would be helpful to consult a lighting professional to ensure the target parameters are accomplished. Also, the individual’s lifestyle, room use, and daily routine should be considered to ensure the individual is exposed to sufficient BL. After BL is installed, it needs to be periodically monitored at room and individual levels to ensure that proper lighting parameters are maintained. The individual’s BPSD also needs to be closely monitored. If an individual demonstrates negative responses to the BL, the lighting may need to be adjusted or discontinued as needed.

### Limitations and Directions for Future Research

To our knowledge, this is the first review that synthesizes evidence specifically on the effect of ambient BL on BPSD in persons with dementia. The key limitation of this review is the widely different lighting design and measurement across studies, making it challenging to compare the results. Additionally, the very different lighting design across studies further complicate the evidence synthesis, including the three aspects of light parameters (i.e., illuminance, CCT, and CS) and different measurement approaches (e.g., vertical versus horizontal, eye-level versus table-height, target versus actual lighting, and in the room versus at participant level).

While this review reveals evidence on the promising effect on depressive symptoms and agitation, this review also identifies several issues and the need for more research work in this field. First, among the nine studies reviewed, there was only one RCT, and most studies had some methodological limitations. More RCT with rigorous design is needed to generate strong evidence. Second, the standard parameters to design, measure, and report BL need to be established. This is a critical foundation for research in this field. Third, while all studies evaluated the effect of BL on BPSD, none of the studies focused on individuals with the specific BPSD of interest. This could result in a flooring effect and underestimate the BL effect. Future studies may further examine the effect of BL targeting people with clinically significant BPSD. Fourth, most studies showed intervention fidelity issues with actual lighting dosages either not clearly reported or much lower than the target dosage. Intervention fidelity should be carefully addressed when planning and implementing the BL and the actual dosage, at both the room and individual level, needs to be closely monitored during the intervention period. Fifth, most studies tested the effect of BL on depressive symptoms and agitation, but only three studies tested its effect on other BPSD. Future research may examine its effect on other BPSD. Especially, it is worth testing the BL that considers CS on other BPSD. Sixth, current ambient BL rarely incorporates natural daylight into their lighting design. While it might not be an easy task, incorporating daylight into ambient BL is a critical step to make BL a sustainable intervention in real life. Finally, the implementation of the lighting intervention needs to be addressed to move forward the intervention into clinical practice.

## Conclusion

This article reviewed 10 articles from nine studies on the effect of ambient BL on BPSD. While not all studies showed positive results, evidence from multiple studies showed that ambient BL approximately targeted at 350–750 lux, 4,500–9,325 K, and CS = 0.375–0.4 for 10–12 hr a day for 4 weeks or longer seems to be beneficial for depressive symptoms and agitation in persons living with dementia. Evidence on other BPSD is too limited to draw conclusions. Future research is needed to further test the effect of ambient BL on BPSD using a more rigorous design while addressing lighting parameters, measurement approaches, and intervention fidelity in future research.
